# Supplementation with Whey Protein, but Not Pea Protein, Reduces Muscle Damage Following Long-Distance Walking in Older Adults

**DOI:** 10.3390/nu15020342

**Published:** 2023-01-10

**Authors:** Marcia Spoelder, Lotte Koopmans, Yvonne A. W. Hartman, Coen C. W. G. Bongers, Merle C. A. Schoofs, Thijs M. H. Eijsvogels, Maria T. E. Hopman

**Affiliations:** Radboud Institute for Health Sciences, Department of Physiology, Radboud University Medical Center, 6500 HB Nijmegen, The Netherlands

**Keywords:** elderly, muscle, protein, randomized controlled trial, plant-based, prolonged exercise, older active adults

## Abstract

**Background**: Adequate animal-based protein intake can attenuate exercise induced-muscle damage (EIMD) in young adults. We examined the effects of 13 days plant-based (pea) protein supplementation compared to whey protein and placebo on EIMD in active older adults. **Methods**: 47 Physically active older adults (60+ years) were randomly allocated to the following groups: (I) whey protein (25 g/day), (II) pea protein (25 g/day) or (III) iso-caloric placebo. Blood concentrations of creatine kinase (CK) and lactate dehydrogenase (LDH), and skeletal muscle mass, muscle strength and muscle soreness were measured prior to and 24 h, 48 h and 72 h after a long-distance walking bout (20–30 km). **Results**: Participants walked 20–30 km and 2 dropped out, leaving n = 15 per subgroup. The whey group showed a significant attenuation of the increase in EIMD at 24 h post-exercise compared to the pea and placebo group (CK concentration: 175 ± 90 versus 300 ± 309 versus 330 ± 165, *p* = *p* < 0.001). No differences in LDH levels, muscle strength, skeletal muscle mass and muscle soreness were observed across groups (all *p*-values > 0.05). **Conclusions**: Thirteen days of pea protein supplementation (25 g/day) does not attenuate EIMD in older adults following a single bout of prolonged walking exercise, whereas the whey protein supplementation group showed significantly lower post-exercise CK concentrations.

## 1. Introduction

The post-exercise inflammatory response, characterized by muscle damage and elevated plasma levels of creatine kinase (CK), is a normal physiological process that is thought to play a vital role in tissue damage repair and enhancing muscle adaptation [1]. Older adults exhibit higher levels of muscle damage and a slower recovery rate upon an exercise bout compared to younger adults [2,3], which may hamper them in pursuing an active lifestyle, accelerating sarcopenia [4].

Daily dietary protein intake is an important factor for muscle repair by enhancing muscle protein synthesis [5,6,7,8]. It has been shown that more than 50% of physically active older adults do not reach the recommended protein intake of 1.2 g/kg/day [9]. Moreover, an increasing number of individuals avoid animal based products in order to reduce their ecological footprint [10]. Studies in young adults have shown that plant-based proteins were able to induce muscle protein synthesis upon digestion, leading to increases in muscle mass after resistance training, comparable to whey protein [11,12,13]. In addition, a reduction in muscle damage was observed in young and middle-aged men after 4 days of eccentric exercise and supplementation both with pea and whey protein [5]. However, no studies have investigated the effect of plant-based protein supplementation on muscle damage markers in older adults who performed a bout of endurance exercise.

This randomized double-blind placebo-controlled trial aimed to assess the effect of pea versus whey protein supplementation in comparison to placebo on exercise-induced increases in muscle damage markers in older adults 1, 2 and 3 days after endurance walking exercise. Secondary outcome measures included differences in changes in muscle soreness, muscle strength and muscle mass among groups after endurance walking exercise. We hypothesized that pea protein supplementation would be able to reduce muscle damage after endurance walking exercise in adults comparable to whey protein supplementation.

## 2. Materials and Methods

### 2.1. Participants

Participants were recruited in July 2021 via the Nijmegen Exercise Study database [14] and advertisements via social media. Individuals aged ≥60 years and capable of walking ≥20 km in a single exercise bout were eligible for inclusion. Exclusion criteria were diabetes mellitus type I and II, being allergic or sensitive for milk proteins or lactose intolerance, a BMI > 30 kg/m^2^, Chronic Obstructive Pulmonary Disease (COPD), renal insufficiency, and an intestinal disease that may influence protein uptake. Moreover, consumption of other freely available protein supplements was not allowed during the study period and participants were requested not to perform prolonged exercises in the 4 days before or after the endurance walking exercise. All participants provided written informed consent prior to any experimental procedure. The study conformed to the principles of the Declaration of Helsinki, was approved by the local Medical Ethical committee (Study-ID: NL77522.091.21) and registered at the Dutch trial registry (#NL9499).

### 2.2. Study Design

**Figure 1:**
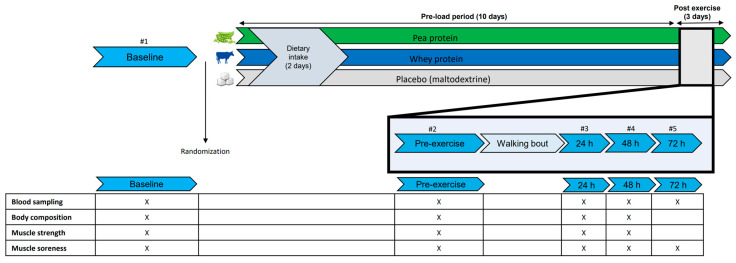
Overview of study timeline and measurements. #1–5 means visit 1–5.

In this randomized double-blind placebo-controlled trial, a total of 47 participants were randomly allocated to a whey protein, pea protein or placebo supplement group. Participants were invited for 5 study visits at the department of Physiology of the Radboud university medical center (Figure 1). Measurements were performed at baseline (i.e., before supplement use), pre-exercise (i.e., after a 10 day pre-load period of supplement use) and three times post-exercise (+24 h, +48 h and +72 h). The exercise bout consisted of a self-selected 20–30 km walk to provoke muscle damage and muscle soreness. Supplement use was continued on the day of the exercise bout and on the three days thereafter.

### 2.3. Supplementation Protocol

Participants were instructed to consume the assigned supplement twice a day for a period of 13 days (10 days prior to the exercise bout and 3 days post exercise). This pre-loading period was chosen as previous studies have demonstrated its importance [15,16], as the lack of pre-loading may prevent the effect of protein supplementation on attenuation of exercise induced muscle damage [17] possibly due to insufficient circulating amino acids. The supplements were provided as a dried powder product (Table 1) in a white container with a twist cap and a measurement spoon inside. Participants were instructed to accurately weigh the correct grams of supplement. Participants were instructed to consume 12.5 g of protein (i.e., 15 g of dry powder) in the morning and 12.5 g of protein in the afternoon/evening (or after exercise). The supplement powder had to be dissolved in a liquid (water, juice) and ingested by the participant. All supplements were provided by NewCare (Waalwijk, The Netherlands) and obtained from DuSart Pharma (Katwijk aan Zee, The Netherlands). Participants were provided with a diary at their first study visit and were asked to report their daily supplement intake throughout the study. The diaries were collected during the last study visit of the participants to assess the adherence of supplement intake. Containers of the supplement were also collected and the remaining content was weighted.

### 2.4. Measurements

#### 2.4.1. Blood Samples

Venous blood was drawn from the antecubital vein during each visit, and serum and lithium heparin plasma samples were stored at −80 °C until further analyses. Analyses were performed by trained technicians using standard operating procedures. CK and LDH levels were measured using a C8000 module C720 clinical chemistry analyzer (Roche, Almere, The Netherlands) to identify muscle damage and tissue damage, respectively.

#### 2.4.2. Habitual Walking Characteristics

The habitual walking activity in the past year (July 2020–July 2021) was assessed at baseline using an online questionnaire. Participants were asked to indicate how frequent they walked per week, the average number of kilometers per walking bout and how many weeks in the past year they performed the reported walking bouts. Hence, the cumulative walking distance was calculated.

#### 2.4.3. Exercise Intervention

Participants were instructed to perform a single walking bout with a minimum of 20 km and a maximum of 30 km. Walking distance, exercise duration and whether participants suffered from physical discomfort were registered directly following the exercise bout.

#### 2.4.4. Muscle Soreness

Muscle soreness was assessed using a Numeric (pain) Rating Scale (NPRS) where participants could mark a pain score between no pain at all (NRS = 0) and extremely painful (NRS = 10). NPRS scores of 1–5 were considered as mild pain, 6–7 as moderate pain and >8 as severe pain [18,19].

#### 2.4.5. Dietary Intake

Food consumption patterns were assessed using an online Dutch tool (i.e., ‘Mijn Voedingscentrum’) [20]. Participants entered their full eating and drinking pattern during 2 days (at least one weekend day) in the pre-load period. Participants were instructed to exclude the supplement from their dietary assessment. The average total energy intake, as well as micro- and macro-nutrient intake of the two recorded days was subsequently calculated and used in the data analyses.

#### 2.4.6. Muscle Strength

Handgrip strength of the right hand was measured with a hydraulic, analogue handheld dynamometer (JAMAR^®^, Chicago, IL, USA). Participants were asked to shortly squeeze the handgrip instrument as hard as they could for three times, with one minute rest in between each measurement [21]. Maximum strength in kilograms was used for analysis.

Leg muscle strength was measured in kilograms with an EN-Dynamic seated leg press (Enraf-Nonius, Rotterdam, The Netherlands). After adjusting the seat and a warm-up, participants had to perform one leg press per fixed step. The weight was increased gradually in seven fixed steps dependent on their body weight to directly determine the 1 repetition maximum (1 RM). There were only 7 attempts to determine the 1 RM to prevent injury and overexerting participants.

#### 2.4.7. Anthropometrics and Muscle Mass

Body height and weight of the participants were measured. Total skeletal muscle mass (SMM) of both legs were estimated with bioelectrical impedance analyses (InBody 770 Body Composition Analyzed, Seoul, Republic of Korea) [22]. Participants were instructed not to eat or drink for 2 h before the measurement and were asked to use the bathroom shortly before their body composition measurement.

## 3. Statistical Analysis

Statistical analyses were performed using SPSS software (IBM SPSS Statistics for Windows, Version 25.0 IBM Corp., Armonk, NY, USA) and graphs were made using Graphpad Prism 9. All continuous variables and the residuals of the variables used in the linear-effects model were visually inspected and tested for normality with the Kolmogorov–Smirnov test. Logarithmic transformations were applied when data were not normally distributed and subsequently the normality was retested. Logarithmic transformations of CK and LDH were used in the statistical analyses in all cases. Data were displayed as mean ± SD or median (interquartile range [IQR]) for parametric and non-parametric continuous variables, respectively. Group differences were analyzed using a One-Way ANOVA or Kruskal–Wallis for parametric or non-parametric continuous variables, respectively. Repeated observations over days, such as CK, LDH and skeletal muscle mass, were analyzed using linear mixed-effects models with compound symmetry as the repeated covariance type in which group and time (e.g., 24 h, 48 h and 72 h upon walking) were treated as independent variables. The pre-exercise values were used as a covariate in the linear mixed model analyses. When significant main effects or interactions were detected, Bonferroni post hoc comparisons were made in case of parametric variables and Mann–Whitney U tests in case of non-parametric variables. Repeated measures analyses for the non-parametric muscle soreness values were performed using a Friedman analysis. The level of significance was set at *p* < 0.05 (two-sided).

## 4. Results

### 4.1. Participants

One participant from the placebo group withdrew from further participation after the baseline measurement due to personal reasons not related to the study protocol. Another participant from the pea supplement group dropped out due to heavy muscle cramps during the walking exercise bout and was therefore unable to complete the minimum walking distance of 20 km. This resulted in 15 participants per subgroup. Participants were predominantly male (80%), aged 70 ± 6 years with a BMI of 24.2 ± 2.8 kg/m^2^. No differences were observed for demographics and anthropometrics across the three supplementation groups (Table 2).

### 4.2. Habitual Dietary Intake and Walking Activity

Total energy intake was 2078 ± 528 kcal and comparable across groups (*p* = 0.57, Table 2). The habitual daily protein intake was 1.13 g/kg/day at baseline and did not differ between the whey, pea and placebo supplemented group (*p* = 0.82). A total of 40% of the participants had a protein intake below 1.0 g/kg/day, but the prevalence was comparable among the three groups. The cumulative walking distance in kilometers in the past year was highly variable across participants (range: 0 to 4680 km/year), with a significantly lower cumulative walking distance for the pea versus whey protein group (*p* = 0.004).

### 4.3. Exercise Bout Characteristic

Participants covered a walking distance of 24.3 ± 4.9 km with a duration of 5.2 ± 1.1 h. Walking distance and exercise duration did not differ across the three groups (*p* = 0.19 and *p* = 0.28, respectively).

### 4.4. Muscle Damage

**Figure 2:**
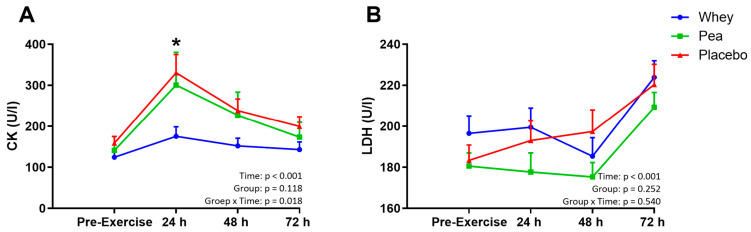
Change in Creatine kinase (CK) (**A**) and Lactate Dehydrogenase (LDH) (**B**) concentrations. Data is presented as mean with Standard Error of the Mean (SEM), while statistical analyses were performed on the logarithmic transformed data with the pre-exercise values as a covariate. * *p* < 0.05 indicates significant group differences at the respective time-point.

The time-dependent exercise-induced increase in creatine kinase (CK) levels were significantly different between the three groups (Pinteraction = 0.018). Peak CK concentrations (+24 h) were significantly lower in the whey (175 ± 90 U/*l*) versus placebo (300 ± 309 U/*l*, *p* = 0.024) and pea supplemented group (330 ± 125 U/*l*, *p* = 0.022, Figure 2A). No differences were observed between the pea- and placebo supplemented group (*p* = 1.00). Correction for the cumulative walking distance in the past year did not alter the observed findings in the statistical analyses (*p* = 0.020). Lactate dehydrogenase (LDH) levels increased over time (*p* < 0.001), and post hoc analyses showed that serum LDH levels at 72 h post-exercise were higher compared to 24 h and 48 h post-exercise (*p* < 0.001, Figure 2B). Nevertheless, no group differences (*p* = 0.25) or interaction effects (*p* = 0.54) were observed in LDH levels.

### 4.5. Muscle Parameters

**Figure 3:**
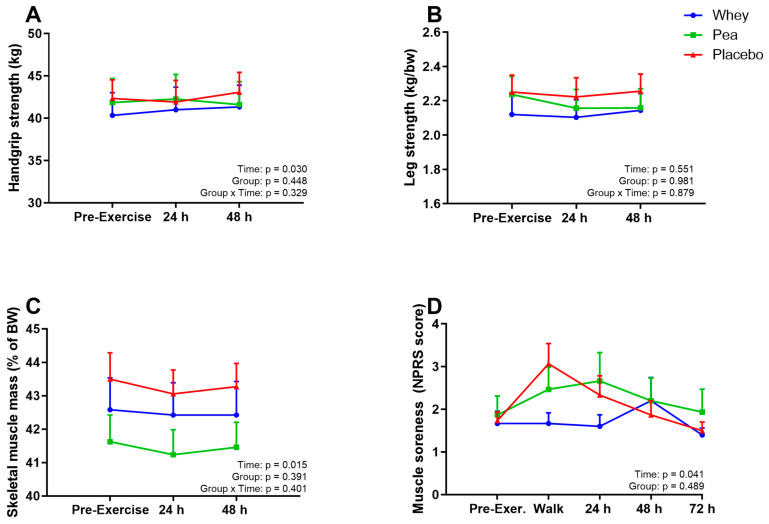
Muscle parameters. Changes in handgrip strength (**A**), leg strength (**B**), skeletal muscle mass (**C**) and muscle soreness (**D**) over time. Data is presented as mean with Standard Error of the Mean (SEM). BW = body weight.

Maximum recorded handgrip strength was not different between groups (*p* = 0.45), but handgrip strength increased over time (*p* = 0.030). This increase did not differ across groups (Pinteraction = 0.33, Figure 3A). Leg strength, corrected for body weight, showed no group differences (*p* = 0.98), nor differences over time (*p* = 0.55, Figure 3B). The skeletal muscle mass of the participants, corrected for body weight (SMM%), was comparable between groups (*p* = 0.39), decreased over time-points (*p* = 0.015), but did not differ across groups (*p* = 0.40, Figure 3C). Muscle soreness increased following exercise (*p* = 0.042), but was comparable between groups (*p* = 0.49, Figure 3D).

## 5. Discussion

The aim of this randomized double-blind placebo-controlled trial was to assess the effect of pea protein and whey protein supplementation versus placebo on exercise-induced increases in muscle damage markers within older adults. We found that long-distance walking exercise provoked muscle damage, with peak CK levels around 24 h post-exercise in physically active older men and women. The CK increase is in line with a previous study who measured CK release in vital elderly after comparable bout of walking exercise [2]. Thirteen days of pea protein supplementation did not impact exercise-induced elevations in CK levels, as reflected by comparable CK responses compared to the placebo group. However, whey protein supplementation attenuated the increase in CK levels at 24 h post-exercise compared to the placebo- and pea protein supplementation group. No differences in LDH levels, muscle strength, skeletal muscle mass and muscle soreness were observed across groups. Findings from this study suggest that thirteen days of pea protein supplementation is not able to attenuate exercise-induced muscle damage in active older adults comparable to whey protein supplementation.

The magnitude of exercise-induced CK elevations was similar in active older adults receiving pea protein supplementation compared to placebo. These findings are contradictory to previous studies using other plant-based proteins, such as soy and oat, as an attenuated exercise-induced increase in CK levels was found compared to the placebo group [15,23]. Potential explanations, apart from the difference in plant protein source, for these discrepant outcomes, may be the higher dosage of the protein that was used (42 gr/day versus 25 gr/day [23]) and the younger age of study participants (<40 years versus >60 years) [15,23] in previous work compared to our study. Older adults are less responsive to the anabolic stimulus of a low dose of amino acid intake than younger individuals [24]. Moreover, the digestibility of protein is also age-dependent, with poorer digestibility rates at older ages [25,26,27,28]. Research on the effects of high dosage plant-based protein supplementation in active older adults would add value to this topic. Alternatively, the type of exercise intervention may have played a role since downhill running and eccentric exercise produce greater CK elevations than prolonged walking exercise [1,2,29,30]. Taken together, 13 days of pea protein supplementation at 25 g per day was insufficient to attenuate the moderate exercise-induced increase in CK levels following prolonged walking in older adults.

In contrast to the pea protein group, an attenuated exercise-induced elevation in CK concentrations was observed for the whey protein group. Whey protein supplementation is known to limit exercise-induced muscle damage [31,32] and its superior effect compared to plant-based protein may relate to differences in the essential amino acid content [33,34,35]. Plant based proteins have a relatively low leucine content (7.1 ± 0.8%) compared to animal-based proteins (8.8 ± 0.7%) [35]. Leucine is known as one of the most important factors of protein synthesis and it also inhibits protein degradation [36]. Previous studies have suggested that every protein supplement serving should contain at least 700 mg of leucine [37]. Given our standardized serving volume for every treatment arm, leucine content was lower for the pea versus whey protein supplement (900 mg versus 1150 mg, respectively). The difference of 250 mg of leucine between these supplements may have contributed to the distinct CK-response following exercise. Fortifying plant-based proteins with leucine may be a promising strategy to limit exercise-induced muscle damage to a comparable extent as is found after whey supplementation. 

The digestibility of whey protein could be another important factor to explain the difference in exercise-induced elevations of CK concentrations compared to pea protein supplementation. Literature about the bio-availability of pea protein is contradictory. In vitro digestibility values are similar between whey and pea protein [38], whereas total amino acid concentrations in humans after digestion seems to be lower for pea compared to whey protein [39]. More research is needed to understand if digestion alters pea protein amino acid availability and transport compared to whey protein. A solution to meet the required amino acid composition in combination with a better digestibility could be to mix different plant-based protein sources to improve the effects [34].

Our study did not show effects of protein or placebo supplementation on changes in serum LDH concentrations, muscle strength, muscle soreness and skeletal muscle mass. The pattern of blood LDH showed a different response to the walking exercise compared to the CK response. This finding in our participants is not in line with most other literature where LDH was used as a marker for muscle damage after short periods of intense exercise (30 min–1.5 h) [40,41,42,43]. However, Rodigues et al. performed a study with fifteen endurance trained male cyclist who performed a 130 km cycling race. In their study they found a similar LDH peak pattern as our study [44]. An explanation for the differences in time of LDH peak can probably be explained by the duration of exercise, since the duration of 130 km cycling is comparable to walk 20–30 km. Most of the studies have shorter exercise bouts while our study has a prolonged relatively low intensity walking bout. The higher handgrip strength at 48 h post-exercise may partially explained by a learning effect [45], but changes over time were small and likely clinically irrelevant. Several studies with protein supplementation varying from 8 weeks to 1 years show an increase in lean body mass, muscle strength and muscle mass for both plant-based and animal-based protein sources in younger and older adults [11,46,47,48,49,50]. This indicates that the 13 days of supplementation that was adopted in our study may not have been long enough to result in changes in muscle strength and skeletal muscle mass. Besides, leg press strength did not change after the walking exercise, suggesting that we either measured the wrong muscles (hamstrings and glutes whereas calf muscles may be more affected by walking exercise), or that the observed muscle damage was not substantial enough to limit muscle strength performance. Differences in the observed muscle damage (i.e., post-exercise CK concentrations) among groups were not reflected in differences in muscle soreness, probably because the magnitude in CK release and differences across groups was only moderate.

Several limitations apply to our study. The protocol used for assessment of 1 RM for leg strength made it difficult to determine 1 RM of participants that reached the maximum of 200 kg or the last attempt. This may have hampered the possible effect of protein supplementation on leg muscle strength. Furthermore, this study also had a relatively small population, which could result in less statistical power and the supplementation period could have been too short to have impact on secondary outcomes. At last, this study had a fixed protein supplementation dose (25 g/day) which increased daily protein intake to >1.0 g/kg/day in 93% of our participants. A personalized protein supplementation dose, based on the individual lean body mass, may therefore be recommended to future studies to achieve a maximal effective dose for all study participants.

## 6. Conclusions

Our randomized double-blind placebo-controlled trial demonstrated that 13 days with 25 g per day of pea protein suppletion did not attenuate exercise-induced muscle damage in older adults compared to placebo. In contrast, the whey protein group showed lower serum CK levels at 24 h post exercise compared to pea protein and placebo group. Furthermore, none of the applied protein supplementation strategies had effect on muscle strength, muscle soreness and skeletal muscle mass, potentially due to the relatively short supplementation period. The effects of long-term plant protein supplementation on exercise induced muscle damage and body composition should be further explored in physically active elderly. Participants may also be exposed to a higher dosage of plant-based protein, whereas the protein quality in terms of digestibility and composition should be taken into account.

## Figures and Tables

**Table 1:** Nutritional composition of whey, pea and placebo supplements.

Nutrient	Whey Protein	Pea Protein	Placebo (Maltodextrin with Lemon Flavor)
Energy (kcal/100 g)	390	382	385
Fat (g/100 g)	8	9	0
Carbohydrate (g/100 g)	6	3.8	94.8
Of which lactose (g/100 g)	6	0	0
Protein (g/100)	81	82	0
Amino Acid content (g/100 g)			
Alanine	4.1	3.6	0
Arginine	2.4	7.2	0
Aspartanic Acid	9.2	9.2	0
Cystine	1.9	0.8	0
Glutamic Acid	12.3	13.2	0
Glycine	1.7	3.2	0
Histidine	1.5	2.0	0
Isoleucine **	4.4	4.0	0
Leucine **	9.2	7.2	0
Lysine	7.3	6.4	0
Methionine	2.1	0.8	0
Phenylalanine	2.8	4.8	0
Proline	5.1	3.6	0
Serine	3.9	4.4	0
Threonine	4.6	3.2	0
Tryptofaan	1.2	0.8	0
Tyrosine	2.4	3.2	0
Valine **	4.5	4.4	0

** Branches Chain Amino Acids (BCAA’s).

**Table 2:** Baseline characteristics of the total group and specified for the whey-, pea- and placebo supplemented group.

	Total Group n = 45	Whey Protein n = 15	Pea Protein n = 15	Placebo n = 15
**Demographics**				
Age (years)	70 ± 6	72 ± 5	69 ± 6	69 ± 6
Male n (%)	36 (80)	12 (80)	12 (80)	12 (80)
**Anthropometrics**				
Body weight (kg)	75.4 ± 13.9	73.9 ± 10.1	75.8 ± 16.3	76.4 ± 15.4
Height (m)	1.76 ± 0.09	1.76 ± 0.07	1.75 ± 0.10	1.76 ± 0.10
BMI (kg/m^2^)	24.2 ± 2.8	23.7 ± 2.2	24.6 ± 3.3	24.4 ± 2.9
Waist-hip ratio	0.97 ± 0.06	0.98 ± 0.07	0.97 ± 0.05	0.97 ± 0.07
**Skeletal muscle mass (%)**	42.5 ± 3.3	42.6 ± 3.9	41.5 ± 3.2	43.4 ± 3.3
**Dietary intake**				
Energy intake (kcal)	2078 ± 528	2153 ± 529	1960 ± 496	2123 ± 570
**Protein intake (g/kg/d)**	1.13 ± 0.33	1.16 ± 0.39	1.09 ± 0.31	1.15 ± 0.31
Protein intake (en%)	22.0 ± 4.7	21.8 ± 5.3	22.1 ± 3.9	22.0 ± 5.1
Number of participants with a protein intake below 1.0 g/kg/d (n (%))	18 (40)	5 (33)	7 (47)	6 (40)
**Walking activity**				
Cumulative walking distance last year (km)	645 [1074]	800 [1784]	280 [520]	820 [988]
Walking bout distance (km)	24.3 ± 4.9	23.8 ± 5.4	23.0 ± 4.4	26.1 ± 4.8
Walking bout duration bout (h)	5.2 ± 1.1	5.2 ± 1.3	4.8 ± 1.0	5.4 ± 1.0

Data are presented as number (with percentage between brackets) of participants, mean ± SD for parametric data or median with the IQR between square brackets for non-parametric data. Body Mass Index (BMI). Thepercentage skeletal muscle mass was calculated by (skeletal muscle mass/the participant’s bodyweight) × 100. (kg = kilogram; m = meter; m^2^ = square meter; g = gram, d = day; en% = energy percentage).

## Data Availability

The datasets generated and/or analyzed during the current study are not publicly available due to the fact that study participants can be identified based on age, sex and finish time, but the pseudonymized dataset is available from the corresponding author upon reasonable request.

## Abbreviations

CK	Creatine Kinase
COPD	Chronic Obstructive Pulmonary Disease
EIMD	Exercise Induced Muscle Damage
LDH	Lactate Dehydrogenase
MPS	Muscle Protein Synthesis
NRS	Numeric (pain) Rating Scale
RM	Repetition Maximum
